# Enhancement of Radiation Effectiveness in Cervical Cancer Cells by Combining Ionizing Radiation with Hyperthermia and Molecular Targeting Agents

**DOI:** 10.3390/ijms19082420

**Published:** 2018-08-16

**Authors:** Marloes IJff, Bregje van Oorschot, Arlene L. Oei, Przemek M. Krawczyk, Hans M. Rodermond, Lukas J. A. Stalpers, H. Petra Kok, Johannes Crezee, Nicolaas A. P. Franken

**Affiliations:** 1 Laboratory of Experimental Oncology and Radiobiology, Amsterdam UMC, University of Amsterdam, Meibergdreef 9, 1105AZ Amsterdam, The Netherlands; m.ijff@amc.uva.nl (M.I.); vobregje@hotmail.com (B.v.O.); a.l.oei@amc.uva.nl (A.L.O.); h.rodermond@amc.uva.nl (H.M.R.); l.stalpers@amc.uva.nl (L.J.A.S.); 2 Center for Experimental Molecular Medicine, Amsterdam UMC, University of Amsterdam, Meibergdreef 9, 1105AZ Amsterdam, The Netherlands; 3 Department of Radiation Oncology, Amsterdam UMC, University of Amsterdam, Meibergdreef 9, 1105AZ Amsterdam, The Netherlands; h.p.kok@amc.uva.nl (H.P.K.); h.crezee@amc.uva.nl (J.C.); 4 Department of Medical Biology, Amsterdam UMC, University of Amsterdam, Meibergdreef 9, 1105AZ Amsterdam, The Netherlands; p.krawczyk@amc.uva.nl

**Keywords:** radiation sensitization, radio enhancement, linear-quadratic model, hyperthermia, PARP1-*i*, DNA-PKcs-*i*, HSP90-*i*

## Abstract

Hyperthermia (HT) and molecular targeting agents can be used to enhance the effect of radiotherapy (RT). The purpose of this paper is to evaluate radiation sensitization by HT and different molecular targeting agents (Poly [ADP-ribose] polymerase 1 inhibitor, PARP1-*i*; DNA-dependent protein kinase catalytic subunit inhibitor, DNA-PKcs-*i* and Heat Shock Protein 90 inhibitor, HSP90-*i*) in cervical cancer cell lines. Survival curves of SiHa and HeLa cells, concerning the combined effects of radiation with hyperthermia and PARP1-*i*, DNA-PKcs-*i* or HSP90-*i*, were analyzed using the linear-quadratic model: S(D)/S(0) = exp − (αD + βD^2^). The values of the linear-quadratic (LQ) parameters α and β, determine the effectiveness at low and high doses, respectively. The effects of these sensitizing agents on the LQ parameters are compared to evaluate dose-dependent differences in radio enhancement. Combination of radiation with hyperthermia, PARP1-*i* and DNA-PKcs-*i* significantly increased the value of the linear parameter α. Both α and β were significantly increased for HSP90-*i* combined with hyperthermia in HeLa cells, though not in SiHa cells. The Homologous Recombination pathway is inhibited by hyperthermia. When hyperthermia is combined with DNA-PKcs-*i* and PARP1-*i*, the Non-Homologous End Joining or Alternative Non-Homologous End Joining pathway is also inhibited, leading to a more potent radio enhancement. The observed increments of the α value imply that significant radio enhancement is obtained at clinically-used radiotherapy doses. Furthermore, the sensitizing effects of hyperthermia can be even further enhanced when combined with other molecular targeting agents.

## 1. Introduction

Cervical cancer is the fourth most common malignant disease in women worldwide [1]. In women with locoregionally advanced cervical cancer, radiotherapy (RT) is the cornerstone of treatment, and is usually combined with cisplatin-based chemotherapy (CT) as a radiosensitizer [2,3,4]. However, some patients do not tolerate cisplatin, because of severe renal or neural toxicity. These patients require other treatment options [5]. Mild hyperthermia (HT), heating the tumor to 39–43 °C for approximately an hour, has been shown to enhance both RT and CT and specifically increases the cytotoxicity of malignant cells [6]. For patients not eligible for CT, HT can be applied in combination with RT to improve tumor control in different types of cancer [7,8]. In several randomized trials in women with cervical cancer, RT applied in combination with HT was shown to improve both tumor control and patient survival [6], to the same extent as chemoradiotherapy [9]. However, in 35% of cases, relapse can still occur. Therefore, the need for improvement of treatment remains. Ionizing radiation induces DNA damage, including single-strand breaks (SSB) and double-strand breaks (DSB), of which DSBs are considered the most lethal. However, most of these lesions are repaired by the various repair mechanisms and modifying these mechanisms may improve the radiation treatment.

**Figure 1:**
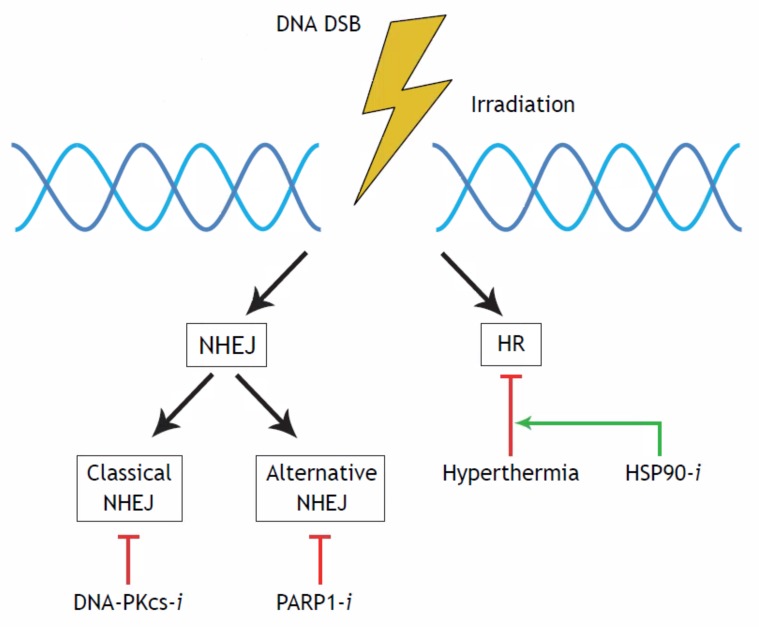
Schematic overview of DNA double-strand break (DSB) repair processes. Treatment options are shown underneath the pathway they act upon. A DSB can be repaired via two major pathways, the Non-Homologous End Joining (NHEJ) or the Homologous Recombination (HR). The NHEJ can be divided into two sub-pathways, the Classical NHEJ (cNEHJ) and the Alternative NHEJ (AltNEHJ). Each pathway can be blocked by specific inhibitors. A DNA-PKcs-*i* can disrupt the Classical NHEJ, A PARP1-*i* the Alternative NHEJ and Hyperthermia can temprarily inactive the HR. A HSP90-*i* can enhance the effectiveness of HT.

There are three distinct pathways involved in DSB repair: Homologous Recombination (HR), classical Non-Homologous End Joining (cNHEJ) and an Alternative Non-Homologous End Joining (AltNHEJ) pathway [10,11,12]. Different agents act in different DNA repair processes and interfering with these pathways can lead to the enhancement of the radiation effects [13,14,15,16], which is shown in Figure 1. Hyperthermia inhibits HR repair as it temporarily degrades the BRCA2 protein [16,17], which explains at least part of its radiosensitizing effect. However, HT is a pleiotropic agent that also affects cells, tissues and organs on multiple other levels [18,19].

To further enhance the radiosensitivity, molecular targeting agents can be used. The number of SSBs per Gy is about 40 times the number of DSBs. Since an unrepaired SSB may progress to a fatal DSB, interfering with SSB repair may also result in therapeutic gains. The molecular targeting drug olaparib acts upon the protein Poly(ADP-ribose) polymerase-1 (PARP1), a protein which is involved in several SSB repair mechanisms [20], but is also involved in the AltNHEJ pathway of DSB repair [21,22,23].

The cNHEJ pathway is responsible for up to 85% of the repair of RT-induced DSBs [23,24]. Important proteins involved are Ku70/80, and the DNA-PK catalytic subunit (DNA-PKcs). The Ku heterodimer binds to the DSB ends, thereby promoting the recruitment and activation of DNA-PKcs [10]. By inhibiting DNA-PKcs, the DSB repair is substantially slowed down leading to an increase in anti-tumor activity of RT [15]. NU7441 is a specific inhibitor of DNA-PKcs. Moreover, it has been shown that hyperthermia may also have an effect on DNA-PKcs [19].

Hyperthermia induces heat shock proteins (HSPs) which may counteract the HT treatment. HSPs are a subgroup of chaperone proteins that strongly respond to increased temperatures to regulate various genes and protect the cells from heat-induced unfolding, inactivation and degradation [25]. One of the members of this group is HSP90, which is of special interest in cancer treatment and HT. This chaperone is evolutionarily conserved and important for the stability of some essential DNA repair factors involved in both HR and cNHEJ [26]. It has been reported that inhibition of HSP90 can enhance the effects of HT on DSB repair [17]. Ganetespib is a new-generation HSP90-inhibitor (HSP90-*i*) that can potentiate HT-induced sensitization to a number of DSB-inducing agents [27].

The clonogenic survival curve is commonly used to estimate and quantify the effect of ionizing radiation and radiosensitizers in tumor cell lines. The radiation dose survival curves can be analyzed using the linear-quadratic (LQ) model: S(D)/S(0) = exp − (αD + βD^2^) [28,29,30]. The LQ model is a mathematical model defining the relationship between linear (α) and quadratic (β) contributions to cell kill and indicates at which RT dose (Gy) cells or tissues will die [31]. The α determines the initial slope of cell survival curves and the effectiveness at low doses of ionizing radiation, while β represents the increasing contribution from cumulative damage, presumably due to the interaction of two or more lesions induced by separate energy depositions [32]. Therefore, by using these parameters, information is obtained on acute and late responses and even additional doses after ionizing radiation-induced breaks can be calculated using the LQ model [29,33].

In this study, both α and β parameters are determined for different combinations of molecular targeting agents and HT. Since HT, olaparib, NU7441 and ganetespib affect different proteins involved in the DNA damage response, it is expected that they may have different effects on the values of the parameters of the linear-quadratic model. Because of this, differences in outcome are expected when combining the therapies. The purpose of this research is to gain an insight into the radio-enhancing effects on cervical cancer cell lines by applying different agents and different combinations of agents, thereby focusing on the effects of the therapies on the different DNA repair processes shown in Figure 1.

## 2. Results

**Figure 2:**
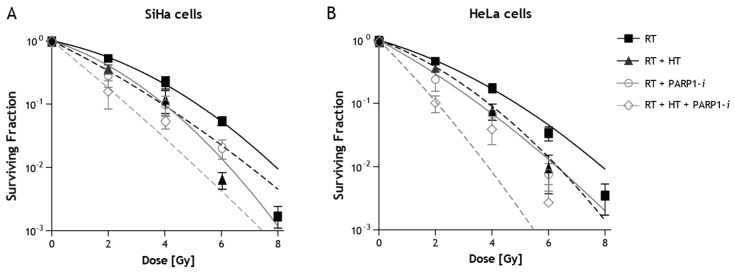
Radiation dose survival curves for SiHa (**A**) and HeLa (**B**) after ionizing radiation (RT) alone, hyperthermia + radiation (HT + RT), radiation + PARP1-*i* (RT + PARP1-*i*) and radiation + hyperthermia + PARP1-*i* (RT + HT + PARP1-*i*).

Radiation dose survival curves of SiHa and HeLa cells after radiation treatment with HT and PARP1 inhibition are depicted in Figure 2. It is clear survival after combined treatments is lower than after radiation alone. Survival curves after combined treatments with HT, HSP90-*i* and DNA-PKcs-*i* are presented elsewhere [14,27]. In Table 1, surviving fractions after 2 Gy only and combined treatments with 2 Gy are given.

**Figure 3:**
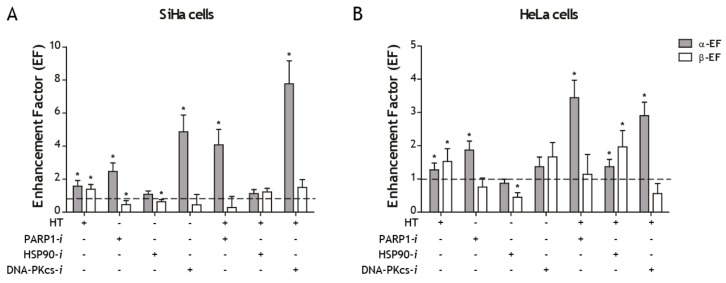
Enhancement factor (EF) of the linear (α) and quadratic (β) parameters after combining radiation with specific inhibitors (HSP90-*i*, DNA-PKcs-*i* and PARP1-*i*) or hyperthermia (HT) and a tri-modality combination in SiHa (**A**) and HeLa (**B**) cells. The value of 1 represents treatment with radiation alone. * Significantly different from radiation alone (*p* < 0.05).

The enhancement factor (EF) of the linear (α) and quadratic (β) parameters for SiHa and HeLa cells after treatment with specific inhibitors (HSP90-*i*, DNA-PKcs-*i* and PARP1-*i*), HT and combined treatment are presented in Figure 3A,B. Values in excess of 1 indicate the presence of sensitization. In SiHa cells, the value of α was significantly increased for the targeting agents olaparib and NU7441 with and without HT. HT alone also showed a significant increase (*p* = 0.001). For the HSP90 inhibitor ganetespib no increase in the value of α was observed, while the value of β was significantly decreased. The value of β was only significantly increased for the combination of RT with HT. The combination of RT with PARP1-*i* was significantly decreased (Figure 3A).

In HeLa cells, the value of α was significantly increased (*p* < 0.05) for the combinations of RT with HT, PARP1-*i* with and without HT and for the triple combinations of RT and HT with DNA-PKcs-*i* or HSP90-*i*. The value of β was significantly increased for the combination of RT with HT, with and without HSP90-*i*, whereas it was significantly decreased for the combination of RT with HSP90-*i* (*p* < 0.05) (Figure 3B).

The non-normalized values of the linear and quadratic parameters for all treatments in both cell lines are summarized in Table 2. The enhancement factors are shown for both α and β (Table 2). The largest number for the α-EF can be observed in the combinational treatment RT + HT + DNA-Pkcs-*i* for SiHa cells and in the combination RT + HT + PARP1-*i* for HeLa cells. However, the standard error is also the biggest in those cases (Table 2).

## 3. Discussion

In this study, the enhanced radiation sensitivity is evaluated from the combined treatment of radiation with or without hyperthermia and with an additional molecular targeting agent. HT alone had a significant sensitizing effect on the linear parameter, α, in both SiHa and HeLa cells, which has also been shown previously in other cell lines [29,31,33]. In SiHa cells, both PARP1-*i* and DNA-PKcs-*i* appeared to be sensitizing agents for low dosages of RT, since the value of the linear parameter was significantly increased (Figure 2). For HeLa cells, the increase of the value of α was significant for PARP1-*i* but not for DNA-PKcs-*i*. For both cell lines the enhancement factor increased when in addition to HT the cells were treated with targeting agents (Figure 2 and Figure 3). This indicates that both sensitizing agents PARP1-*i* and DNA-PKcs-*i* can be used to enhance the effects of HT for low dosages of RT. The value of the quadratic parameter β, was only significantly increased for HeLa cells treated with HT and HSP90-*i*. In all other cases the value of β was unchanged or even significantly lower than for radiation alone.

Doses up to 3 Gy are commonly used in the clinic in fractionated applications, implying that the effects of these tri-modality treatments studied here, might be effective in the clinic to improve tumor control [31]. It is of interest to assess whether the changes in the values of the LQ parameters can be interpreted in terms of DNA DSBs and in relation to DSB repair. An increase of the values of α and β, indicating a higher level of cell death and indeed increased numbers of DSBs, as studied with γH2Ax foci, have been found in cervical cancer and breast cancer cells when ionizing radiation is combined with DNA repair targeting agents [14]. The increased sensitivity for irradiation can be explained by the effects on the DNA repair pathways, as shown in Figure 1.

A functional hierarchy exists between the three repair pathways, in which cNHEJ dominates over the other two pathways [34]. Several proteins are involved in cNHEJ, and a lack of any of these core proteins creates a severe DNA repair defect and increased sensitivity to irradiation [34]. DNA-PKcs is one of the proteins involved in the end processing step in the cNHEJ pathway, so by inhibiting this protein the tumor cell is no longer able to rely on this mechanism, leading to an accumulation of DSBs. This effect is enhanced when combined with HT, since this combination increases radiation sensitivity by inhibiting both cNHEJ as well as HR.

PARP1 is involved in both the AltNHEJ DNA DSB repair pathway, as well as in the DNA SSB repair pathway [13,35]. Normally, the AltNHEJ pathway, which is primarily responsible for the formation of translocations [23], is suppressed by the cNHEJ pathway. However, we show that inhibiting AltNHEJ, especially in combination with HT, also enhances radiation sensitivity at low dosages (Figure 2 and Figure 3). Probably more DSBs arise as compared to radiation alone or duo treatments due to repair inhibition by HT but also due to the conversion of SSB to DSB after PARP1 inhibition.

The α enhancement factor in both SiHa and HeLa cells was not significantly increased for the combinations of RT with HSP90-*i*. An increase in the enhancement factors was observed only when combined with HT, indicating that HSP90-*i* can be used to improve the inhibitory effects of HT on HR (Figure 2 and Figure 3). This is in agreement with previously published data [27].

By combining all four treatments, all DSB repair pathways would be inhibited. Since the AltNHEJ pathway is suppressed by cNHEJ, it can only rely on this pathway when the other pathways are not able to repair the lesion. HT is used in the clinic in combination with RT [36] or CHT [37,38] and in phase III trials it has proven to be an effective and less toxic alternative treatment for chemoradiation [9]. Olaparib is already used in the clinic in combination with cisplatin to treat different types of cancer [39]. Several DNA-PKcs inhibitors are currently used in phase I trials for advanced solid tumors [40,41]. Therefore, it might be beneficial to see how this combinational treatment can be further enhanced. For the future, it would be interesting to test a four-modality combinational treatment in vitro comparing effects on tumor tissue with healthy tissue. Eventually, it would be good to see what the effects will be in vivo as well.

## 4. Methods

In vitro experiments for radiation combined with hyperthermia and another agent have been published in earlier works [14,27,31,42]. Enhancement of radiation by hyperthermia and targeting drugs is expressed by increases in either the value of α and/or the value of β of the LQ formula. This reflects an increase in radiation sensitivity as a result of the combined treatment. This methods section describes the combinational treatments and analysis of the data.

### 4.1. Linear-Quadratic (LQ) Model

The linear-quadratic formula: S(D)/S(0) = Exp − (αD + βD^2^) was used for analysis of cell survival curves obtained from combined treatments in which D is the radiation dose, S(D) is the surviving fraction after radiation dose D, S(0) is the surviving fraction after dose 0 and α and β are parameters that determine the radiation sensitivity. These values depend on the culture conditions and the presence of radiosensitizing agents [28]. The enhancement factors (EF) for lethal and sublethal damage were calculated from the values of the LQ parameters:

α-EF = enhancement factor α. This is defined as the ratio of the value of α after combinational treatment and the value of α for radiation alone.

β-EF = enhancement factor β. This is defined as the ratio of the value of β after combinational treatment and the value of β for radiation alone.

### 4.2. Cell Cultures

The human cervical cell lines SiHa and HeLa were used, as obtained from the American Type Culture Collection (ATCC, Manassas, VA, USA). The cells were cultured at 37 °C 5% CO_2_ in Eagle’s minimum essential medium containing 10% fetal bovine serum (FBS) and 2 mM glutamine. The medium was supplied from Gibco-BRL life technologies, Breda, The Netherlands.

### 4.3. Irradiation

Radiation treatments were performed with multiple radiation doses (0, 2, 4, 6 and 8 Gy) of γ-rays from a ^137^Cs source at a dose rate of about 0.5 Gy/min.

### 4.4. Hyperthermia

Cells were treated with hyperthermia at 42 °C for 1 h immediately prior to irradiation. 42 °C for 1 h was chosen as this temperature is often used in the clinic. Hyperthermia was carried out by submerging the 6-wells plates in a thermostatically-controlled water bath. The temperature was checked in parallel plates and the desired temperature (±0.1 °C) was reached in approximately 5 min.

### 4.5. Molecular Targeting Agents

PARP1 inhibition was induced using olaparib. Cells were treated continuously with 5 µM olaparib (Lynparza^®^, AstraZeneca, Cambridge, UK, dissolved in Eagle’s minimum essential medium) 4 h prior to the HT treatment and/or RT. For HSP90 inhibition, cells were treated with 3 nM ganetespib (STA-9090, Synta Pharmaceuticals, West Conshohocken, PA, USA, dissolved in DMSO) 24 h before the start of HT treatment and/or RT.

DNA-PKcs was inhibited using a specific inhibitor (NU7441, Selleckchem, Munich, Germany). NU7441 was dissolved in DMSO as 10 mM stock, further diluted in PBS to 1 mM and added to culture medium at a final concentration of 1 μM.

### 4.6. Clonogenic Assay

Clonogenic assays in HeLa and SiHa cells were conducted as described by Franken et al. (2006) [43]. Cells were plated in 6-wells plates at different densities before treatment. After receiving treatment, cells were incubated for 10 days to form colonies. Surviving colonies were fixated and stained with a glutaraldehyde-crystal violet solution and counted manually. Experiments were carried out at least 4 times.

### 4.7. Statistical Analysis

Means and standard deviations (SD) were calculated for all data points from at least three different experiments. The Student’s *t*-test was used to analyze and compare the means. A significant difference was considered when *p* < 0.05.

## 5. Conclusions

Low RT doses are often used in fractionated-radiation treatment in the clinic. In our results a significant increase of the value of α was obtained in most cases using HT or targeting agents, indicating radio enhancement at clinically-used RT doses. HT and both targeting agents, DNA-PKcs-*i* and PARP1-*i*, had a larger enhancing effect on the linear parameter α and thus contributed more to enhanced radiosensitivity at low RT doses compared to HSP90-*i*. Furthermore, the sensitizing effects of HT and the value of α were demonstrated to be even further increased when HT treatment was combined with one of the sensitizing agents. The HR pathway gets inhibited by HT, and when using DNA-PKcs-*i* and PARP1-*i* as well, the NHEJ pathways are also inhibited, leaving no remaining pathway to repair DNA damage, thus yielding a potent combinational therapy.

## Figures and Tables

**Table 1:** Values of surviving fractions after 2 Gy only and combined treatments 2 Gy with hyperthermia (HT), PARP1-*i*, HSP90-*i* and DNA-PKcs-*i*.

Cell Line	Sensitizing Agent	Surviving Fraction
SiHa	RT	0.53 ± 0.08
SiHa	RT + HT42	0.36 ± 0.10
SiHa	RT + PARP1-*i*	0.28 ± 0.09
SiHa	RT + HT42 + PARP1-*i*	0.16 ± 0.07
SiHa	RT + HSP90-*i*	0.58 ± 0.04
SiHa	RT + HT42 + HSP90-*i*	0.51 ± 0.05
SiHa	RT + DNA-PKcs-*i*	0.12 ± 0.03
SiHa	RT + HT42 + DNA-PKcs-*i*	0.03 ± 0.00
HeLa	RT	0.46 ± 0.03
HeLa	RT + HT42	0.36 ± 0.09
HeLa	RT + PARP1-*i*	0.24 ± 0.08
HeLa	RT + HT42 + PARP1-*i*	0.10 ± 0.03
HeLa	RT + HSP90-*i*	0.56 ± 0.03
HeLa	RT + HT42 + HSP90-*i*	0.33 ± 0.04
HeLa	RT + DNA-PKcs-*i*	0.35 ± 0.06
HeLa	RT + HT42 + DNA-PKcs-*i*	0.16 ± 0.02

**Table 2:** Values of the parameters α and β and their ratio for radiotherapy alone and in combination with the different sensitizing agents.

Cell Line	Sensitizing Agent	α (Gy^−1^)	β (Gy^−2^)	α-EF *	β-EF *
SiHa	RT	0.21 ± 0.04	0.05 ± 0.01	1.00 ± 0.00	1.00 ± 0.21
SiHa	RT + HT42	0.32 ± 0.07	0.07 ± 0.01	1.56 ± 0.45	1.38 ± 0.28
SiHa	RT + PARP1-*i*	0.51 ± 0.05	0.02 ± 0.01	2.46 ± 0.51	0.45 ± 0.24
SiHa	RT + HSP-*i*	0.22 ± 0.02	0.03 ± 0.00	1.07 ± 0.21	0.62 ± 0.13
SiHa	RT + DNA-PKcs-*i*	1.00 ± 0.11	0.02 ± 0.03	4.85 ± 1.02	0.43 ± 0.64
SiHa	RT + HT + PARP1-*i*	0.84 ± 0.12 **	0.01 ± 0.03	4.06 ± 0.94 **	0.25 ± 0.68
SiHa	RT + HT + HSP-*i*	0.23 ± 0.03	0.06 ± 0.01 ***	1.12 ± 0.24	1.21 ± 0.22 ***
SiHa	RT + HT + DNA-PKcs-*i*	1.60 ± 0.02 **	0.07 ± 0.02	7.77 ± 1.40 **	1.49 ± 0.48
HeLa	RT	0.30 ± 0.04	0.04 ± 0.01	1.00 ± 0.18	1.00 ± 0.00
HeLa	RT + HT42	0.38 ± 0.04	0.06 ± 0.01	1.27 ± 0.21	1.53 ± 0.39
HeLa	RT + PARP1-*i*	0.56 ± 0.04	0.03 ± 0.01	1.88 ± 0.27	0.75 ± 0.27
HeLa	RT + HSP-*i*	0.26 ± 0.02	0.02 ± 0.00	0.87 ± 0.12	0.44 ± 0.14
HeLa	RT + DNA-PKcs-*i*	0.41 ± 0.07	0.06 ± 0.01	1.37 ± 0.29	1.67 ± 0.43
HeLa	RT + HT + PARP1-*i*	1.03 ± 0.09 **	0.04 ± 0.02	3.46 ± 0.53 **	1.14 ± 0.60
HeLa	RT + HT + HSP-*i*	0.41 ± 0.04 ***	0.07 ± 0.01 ***	1.37 ± 0.22 ***	1.97 ± 0.49 ***
HeLa	RT + HT + DNA-PKcs-*i*	0.87 ± 0.06 **	0.02 ± 0.01	2.91 ± 0.41 **	0.56 ± 0.30

* EF = Enhancement Factor; ** significantly different from both duo modality treatments; *** significantly different from RT + HSP-*i*.
